# Impact of Heart Failure Team on Inpatient Rapid Sequencing of Heart Failure Therapy

**DOI:** 10.3390/jcdd12020050

**Published:** 2025-01-28

**Authors:** Zhongrui Zhou, Khalid Kardas, Ying Xuan Gue, Ali Najm, Anas Tirawi, Rachel Goode, Robert Frodsham, Rory Kavanagh, Archana Rao, Rebecca Dobson, David Wright, Matthew Kahn

**Affiliations:** 1 School of Medicine, Faculty of Health & Life Sciences, University of Liverpool, Liverpool L69 3GE, UK; johnzhou@doctors.org.uk (Z.Z.); khalidkardas@doctors.org.uk (K.K.); ying.gue@lhch.nhs.uk (Y.X.G.); 2 Liverpool Heart & Chest Hospital, Liverpool L14 3PE, UK; ali.al-zubaidi@lhch.nhs.uk (A.N.); anas.tirawi@lhch.nhs.uk (A.T.); rachel.goode@lhch.nhs.uk (R.G.); robert.frodsham@lhch.nhs.uk (R.F.); rory.kavanagh@lhch.nhs.uk (R.K.); archana.rao@lhch.nhs.uk (A.R.); rebecca.dobson@lhch.nhs.uk (R.D.); david.wright@lhch.nhs.uk (D.W.); 3 Liverpool Centre for Cardiovascular Science at University of Liverpool, Liverpool John Moores University and Liverpool Heart and Chest Hospital, William Henry Duncan Building, 6 West Derby Street, Liverpool L7 8TX, UK; 4 Department of Cardiovascular and Metabolic Medicine, Institute of Life Course and Medical Sciences, University of Liverpool, Liverpool L69 7ZX, UK

**Keywords:** heart, failure, inpatient, team, rapid, sequencing

## Abstract

The management of heart failure (HF) has undergone a paradigm shift from conventional stepwise methods of initiation and the up-titration of HF therapy towards an early, more intensive initiation of pharmacotherapy to improve the prognosis. The aim of this study was to compare the outcomes of patients at the Liverpool Heart and Chest Hospital (LHCH), with new diagnosis of HF, who were reviewed by the inpatient heart failure team (HFT), compared to patients that were not reviewed. A retrospective review of the electronic records of patients admitted with a new diagnosis of HF to the LHCH from May to December 2023 was performed. Admission drugs were similar, apart from betablockers, which were more frequent in the non-HFT group (58% vs. 24.2%; *p* = 0.002). The length of inpatient stay was longer in the HFT group (median 5.5 days vs. 3 days; *p* = 0.001) and more likely to be on all four pillars of HF medical therapy (96.8% vs. 0; *p* < 0.001) within 30 days of discharge. The 30-day and 6-month mortality outcomes were not significantly different. Patients reviewed by the HFT were significantly more likely to receive the four pillars of HF therapy within 30 days of their diagnosis compared to their counterparts at the expense of a longer length of stay.

## 1. Introduction

Heart failure (HF) is a complex medical syndrome resulting in the impairment of ventricular filling or the ejection of blood, which carries significant medical and psychological complication to the patient, which are associated with high levels of multisystem morbidity and mortality rates [1,2]. It is classified by left ventricular ejection fraction (LVEF), with heart failure with a reduced ejection fraction (HFrEF), defined as LVEF ≤ 40%, being the class that is most prevalent globally [3]. HF remains an immense burden on the NHS in the UK, accounting for 2% of the total NHS budget and 5% of all emergency admissions [4]. It is estimated that over one million patients in the UK have HF, with over 200,000 new diagnoses every year; this figure is expected to increase due to the ageing population [5].

Advancements in medical therapy have significantly improved the prognosis of HF patients [6]. The foundational therapy of HFrEF comprises of the four pillars, namely, betablockers, sodium glucose co transporter 2 inhibitors (SGLT2i), mineralocorticoid receptor antagonists (MRA), and renin–angiotensin–aldosterone system (RAAS) modulators in the form of angiotensin-converting enzyme inhibitor (ACE-i), angiotensin receptor blocker (ARB), or angiotensin receptor–neprilysin inhibitor (ARNI) [7,8]. Early initiation and therapy following the diagnosis of HFrEF is linked with improved outcomes, which has led to the paradigm shift from the conventional stepwise approach to an accelerated rapid-sequencing strategy, with the aim to establish patients on all four pillars in the shortest time safely [9]. This was further supported by the recent STRONG-HF study, which showed that an intensive strategy was safe and resulted in improved outcomes in patients with HFrEF [10]. The inpatient heart failure team (HFT) could facilitate the initiation of an intensive strategy of starting foundational therapy, with subsequent follow-up after discharge from the hospital; it currently has a Class 1A recommendation from the European society of Cardiology [11].

The prognosis of HF patients within the United Kingdom remains poor, with a 5 year survival rate at 48.2%; statistics from the National Heart Failure Audit UK 2022 showed that inpatient mortality was 9.2%, and only 65% of hospitals achieved the recommended rate of specialist reviews [12,13]. We aim to explore the role and impact of an inpatient HFT within a single tertiary centre based in the UK.

## 2. Materials and Methods

This was a single-centre, retrospective cohort study conducted at the Liverpool Heart and Chest Hospital (LHCH) in the UK. The study included all patients admitted over a one-year period with a documented new left ventricular ejection fraction (LVEF) of ≤40%. Patients with a pre-existing diagnosis of heart failure on admission or patients with a LVEF of ≤40% were excluded from the study. Within the LHCH, referral for review by the HFT is dependent on the parent treating team and is commonly prompted by the presence of an HFrEF requiring the optimisation of HF therapy or an advanced HF input. Patients were categorised into the following two groups: whether or not they had been reviewed by the inpatient HF team, namely, the HF specialist nurses or doctors who can initiate HF treatment.

Data collection was performed by two independent investigators using patient hospital case notes on the electronic patient record as the primary data source. Data were collected separately for each group and subsequently compared in the final analysis. The following variables were recorded: gender, age, partial postcode, new diagnosis status, heart failure aetiology, revascularisation therapy, outpatient follow-up date, length of stay, admission medications and dosages, admission devices, discharge medications and dosages, discharge devices, inpatient and outpatient LVEF with corresponding dates, and change in the New York Heart Association (NYHA) class from admission to outpatient follow-up. Aetiology was separated into ischaemic cardiomyopathy (ICM) and non-ischaemic cardiomyopathy (NICM). ICM is defined as reduced systolic function secondary to myocardial ischaemia, often due to coronary artery disease (CAD) or acute coronary syndrome (ACS), eventually leading to HF [14,15]. NICM is defined as a range of dilated, hypertrophic, and arrhythmogenic cardiomyopathies in the absence of abnormal loading conditions, such as hypertension, valve disease, or ischaemic aetiology such CAD or ACS [16,17,18]. The primary outcome of interest was the initiation of the 4 pillars of HF therapy within 30 days of admission. Other outcomes of interests included readmissions and mortality (30 days and 6 months), and the length of stay.

Data are presented as the median (interquartile range), as they are non-normally distributed. Dichotomous variables were compared using Fisher’s exact test. Paired comparisons of continuous variables between groups were evaluated with the Wilcoxon ranked sum test and Kruskal–Wallis rank test. Statistical significance was defined as *p* < 0.05. Analyses were performed with Stata version 15.1 (StatCorp, College Station, TX, USA).

## 3. Results

### 3.1. Patient Characteristics

Data extracted from electronic health records from the LHCH over a 7-month period between May 2023 to December 2023 are shown in Table 1. A total of 91 patients were admitted with a new diagnosis of HF with an LVEF ≤ 40%, of which 68.1% (*n* = 62) were reviewed by the HFT. The mean age of those reviewed by the HFT was 64 years, with 80.7% (*n* = 50) being male. There was no statistically significant difference between age and gender between patients reviewed by the HFT and those that were not reviewed. The LVEF was significantly lower in the patient cohort reviewed by the HFT compared to those not reviewed (32.0 vs. 37.5; *p* < 0.001). The most common aetiology among both cohorts was ischaemic cardiomyopathy, accounting for 75.9% of HF patients not seen by the HFT and 69.4% of patients reviewed by the HFT, with no significant differences.

### 3.2. Medical Therapy

The patient cohort reviewed by the HFT had a lower prescription rate of BB on admission compared to the patient cohort that was not reviewed (24.2% vs. 58.7%; *p* = 0.002), but there were no significant differences for the prescription rates of the other foundational drugs on admission (Table 2). There was a statistically significantly higher proportion of patients in the group reviewed by the HFT who were discharged on RAAS modulation, MRA, and SGLT2is, and, consequently, a higher proportion were on all four pillars of heart failure medication within 30 days of diagnosis (96.8% vs. 0%; *p* < 0.001).

### 3.3. Length of Inpatient Stay, Mortality, and Readmission

The mean length of stay was significantly longer in those that were reviewed by HFT (5.5 days vs. 3 days; *p* = 0.001). There was no significant difference in outcomes at both 30 days and 6 months post discharge in both patient cohorts although there were low event rates (Table 3).

## 4. Discussion

In this single-centre study, the patients that were reviewed by the inpatient HFT had a significantly higher proportion of being on the four pillars of evidence-based HF medical therapy at both discharge and 30 days post-discharge when compared to the patients who were not reviewed by the HFT. This highlights the impact that the HFT has on the rapid initiation of prognostic therapy in patients with newly diagnosed HFrEF, and supports the effort to change our practice in HF management from the conventional slower initiation and up-titration to the rapid sequencing approach [9]. Current existing evidence supports the outcomes that favour patients with a new diagnosis of HF who have been reviewed by the inpatient HFT, including lower readmission rates, mortality, and better symptomatic control [19,20,21]. As supported by the recent STRONG-HF study, the rapid initiation and up-titration of pharmacological therapies have been shown to be safe, have significantly improved the quality of life, and have reduced the risk of death or being readmitted for heart failure across a range of HF aetiologies, both ischaemic and non-ischaemic, across all patient age groups and patients with differing comorbidities [10,22]. Age is commonly a barrier to intensive pharmacological therapy, particularly with older adults, where it has been shown in studies that the titration was poorer [23,24]. A recent subgroup analysis has shown that there was no difference in the all-cause death and HF readmission between age groups, i.e., above and below 65 years old, even though older patients have a smaller benefit in quality of life [22]. These studies highlight the prognostic implications that intensive therapy has on older adults, and ages should not be used as a barrier to early high-intensity pharmacological therapy.

Apart from providing inpatient advice in the management of HF, as recommended by NICE [25], our study showed the value of the inpatient HFT in supporting this approach, which could further improve the prognosis in acute HF patients. Data from the recent national heart failure audit (NHFA) have shown that 82% of the patients were seen by a HF specialist during their admission, although the target of 80% is only achieved by 62% of the hospitals within the UK [26]. The audit also confirmed the results of our study, in which patients who had HF specialist input were more likely to receive prognostic therapy [26].

Although detailed data regarding the reason for longer inpatient stay was not extracted in our study, it raises the theory that patients seen by the heart failure team were in a poorer clinical condition, and therefore were more likely to warrant an HFT referral compared to those that were not seen. A literature review showed that often prolonged hospital stays in HF patients can be associated with complex clinical symptoms at admission, and may also be due to differences and variations in clinical practice [27]. Our data showed a significantly longer stay with patients being reviewed by the HFT, which is consistent with the NHFA [26]. The longer length of stay provides the opportunity to ensure appropriate medical therapy to be initiated, but the optimal stay is unknown. The underlying aetiology can have a significant impact on the duration of admission, and can subsequently result in higher costs [28]. Higher levels of NT-proBNP and troponin can be directly correlated to prolonged hospital admission; therefore, the admission length could be considered as a surrogate for the HF severity. Given that our data collection did not focus on these factors, this can be considered as a future scope for study [29].

Further studies also support inpatient HFT providing a more comprehensive review of heart failure medication, and better prescription rates and adherence; in turn, this also led to better patient outcomes and prognosis [20,30,31]. Recently, there has been a paradigm shift, with studies proposing faster sequencing methods of HF medical therapy compared to conventional slower sequencing methods [32]. Further studies have shown that rapid sequencing, which prioritises the early and simultaneous initiation of all four pillars of HF medical therapy before full-dose titration in HFrEF patients, significantly improves the prognosis by reducing hospitalisation and mortality [33,34,35]. The sequence of therapy initiation is also an important aspect of rapid sequencing, which was not explored within our study. Recognising which of the four pillars can be initiated first based on patient characteristics, such as heart rate, blood pressure, and renal function, is an important aspect of ensuring that patients tolerate optimal therapies. Specialist input combined with the rapid sequencing method of medical therapy at the LHCH is the most likely reason for why patients reviewed by the HFT were significantly more likely to receive prognosis-improving medical therapy [36]. However, despite the strong international evidence supporting medical therapies that improve patients in the short term, the data from our own study did not show any significant improvement in mortality, morbidity, or readmission rates in the short term [37,38]. This is likely due to the small numbers and the short follow-up durations of our patients

Interestingly, in the cohort not reviewed by the HFT, none of the patients were on the four pillars of HF therapy within 30 days post-discharge. The missed opportunity for inpatient initiation of HF therapy, which often leads to further delays in achieving optimal medical therapy, further highlights the value of inpatient specialist HFTs. The findings from our single centre retrospective study build upon a current pool of literature that is in favour of a heart failure specialist team to directly manage patients with a new diagnosis of HF with reduced ejection fraction, thereby supporting the Class 1A ESC recommendation [11,39].

One major limiting factor is the structure of the EHR that the investigators extracted the data from; the LHCH is a regional centre for subspecialised cardiology and receives referrals across multiple hospitals in Northwest England. Therefore, patients who are discharged and deteriorate post-discharge, requiring readmission for the management of HF, would often not be readmitted to the LHCH if there was no clinical indication, and instead would be readmitted to a local hospital. Therefore, we are unable to accurately extract data from our EHR regarding readmission rates. Furthermore, due to this, we are also unable to accurately assess the difference in outcomes between the higher levels of prescription rates seen in patients reviewed by the HFT. Therefore, a follow-up study should focus on extracting data from local hospitals regarding the same patients. On a similar note, the small numbers might limit the conclusions drawn on the impact of the outcomes in our patients.

Secondly, the length of stay is not reflective of the total length of stay, as patients are often transferred from a periphery hospital, where they might have had inpatient stay for several days prior to the transfer. This could explain the differences between our median length of stay with the NHFA data.

Left ventricular reverse remodelling (LVRR) is a known phenomenon that is characterised by improvement in systolic and diastolic function [40]. The numerical definition for LVRR is complex, with significant heterogeneity among studies, combining different ranges of increased LVEF, decreased left ventricular end diastolic volume (LVEDV), and decreased left ventricular end-systolic volume (LVESV) [41,42,43]. Furthermore, left atrial reverse remodelling is a known positive prognostic factor for HF patients [44,45]. Various single-centre studies measured LARR through varying reductions in the left atrial volume (LAV) [46]. Optimised medical therapy using the four pillars of HF therapy is known to significantly improve patient prognosis, outcomes, and the rate of LVRR as well as LARR [41,46,47,48,49]. Hence, the values for the LAV and LV volume could be used in the future as surrogate measures of the effectiveness of the HF therapy. These biomarkers were not systematically assessed during admission and at follow-up; therefore, further conclusions which could have resulted from analysis cannot be drawn. This highlights another area of improvement within our service.

Lastly, as the LHCH is a tertiary referral centre, patients who are older or have more comorbidities deemed unsuitable for invasive interventions would not be transferred; hence, the mean age within our cohort is lower than expected.

Overall, this was a retrospective observational study and not a randomised controlled trial; therefore, it provided little insight into the casual association between variables. Given that our data extraction did not focus on investigations, clinical examinations, and imaging, we are unable to fully understand the causal relationship between patients reviewed by the HFT and prolonged hospital admission. It is likely that there is maybe a case of a more complex clinical presentation being a confounding variable, meaning a longer patient stay in a cohort reviewed by the HFT. Furthermore, another confounding variable could be that an HFT review and referral warrants a longer hospital stay to up-titrate medication and correctly implement longer-term management strategies.

## 5. Conclusions

This study highlighted the effect that an inpatient HFT review had on the rates of prescription of the four pillars of heart failure management in patients with HFrEF. Our results show that the patients reviewed by an inpatient HFT were more likely to be started on the four pillars of HF management, although this did not result in a difference in outcomes, probably due to the limitations described above. Therefore, further investigation is warranted to gain a better understanding of the long-term effects that impact on patient outcomes. Further studies should focus on collecting comprehensive follow-up information across multiple hospital sites to assess the long-term effects, such as hospital readmission and mortality rates.

This study reiterates the importance and value of inpatient specialist HFT review, as it can aid in optimising medical management.

## Figures and Tables

**Table 1:** Baseline characteristics of the patients included in this study.

	Patients Not Reviewed by the HF Team (*n* = 29)	Patients Reviewed by the HF Team (*n* = 62)	*p*-Value
Age	66 (62–73)	64 (52–72)	0.079
Male	22 (75.9)	50 (80.7)	0.59
Left ventricular ejection fraction (%)			
	37.5 (34–40)	32 (25–35)	<0.001
HF Aetiology Ischemic cardiomyopathy (ICM)			0.08
	22 (75.9)	43 (69.4)	
ICM patients undergoing revascularisation	17 (58.6)	31 (73.8)	
Non-ischemic cardiomyopathy	1 (3.4)	12 (19.3)	
Others	6 (20.7)	7 (11.3)	

Values are the median (IQR) or n (%). HF—heart failure

**Table 2:** Admission and discharge medical therapy for the patients studied.

	Patients Not Reviewed by the HF Team (*n* = 29)	Patients Reviewed by the HF Team (*n* = 62)	*p*-Value
Admission Drugs			
Beta-blockers	17 (58.7)	15 (24.2)	0.002
RAAS modulation	12 (41.4)	18 (29.0)	0.339
MRA	3 (10.3)	3 (4.8)	0.379
SGLT2i	3 (10.3)	6 (9.7)	1.000
Diuretic	5 (17.2)	4 (6.5)	0.137
Discharge Drugs			
Beta-blockers	28 (96.6)	62 (100)	0.319
RAAS modulation	13 (44.8)	61 (98.4)	<0.001
MRA	10 (34.5)	61 (98.4)	<0.001
SGLT2i	3 (10.3)	62 (100)	<0.001
Diuretic	11 (37.9)	25 (40.3)	1.000
Documented reason for not starting heart failure treatment	4 (13.8)	2 (100)	
On the four pillars of HF therapy within 30 days of diagnosis	0 (0)	60 (96.8)	<0.001

Values are the median (IQR) or n (%). HF—heart failure; MRA—mineralocorticoid antagonist; RAAS—renin angiotensin aldosterone system; SGLT2i—sodium-glucose co-transproter-2 inhibitor.

**Table 3:** Length of inpatient stay and outcomes for the patients included in the study.

	Patients Not Reviewed by the HF Team (*n* = 29)	Patients Reviewed by the HF Team (*n* = 62)	*p*-Value
Length of stay (days)	3 (2–6)	5.5 (3–9)	0.001
Outcomes (30 days)			
Mortality	0 (0)	0 (0)	
HF readmission	1 (3.5)	0 (0)	0.319
Outcomes (6 months)			
Mortality	0 (0)	3 (4.8)	0.549
HF readmission	1 (3.5)	1 (1.6)	1.000

Values are the median (IQR) or n (%). HF—heart failure

## Data Availability

The data presented in this study are available on request from the corresponding author due to patient confidentiality.
